# The Study of the Internal Secretions

**Published:** 1917-01

**Authors:** 


### The Study of the Internal Secretions.

For some months the readers of the TEXAS MEDICAL JOURNAL have been treated to a very interesting series of articles on the "Diagnosis of Internal Secretory Disorders," by Dr. Harrower of Los Angeles, and the series has aroused quite a little interest amongst our readers.

We were recently informed by Dr. Harrower that a new scientific society has been started called "The Association for the Study of the Internal Secretions," the principal objects of which are to broaden our still somewhat limited knowledge in this field, and establish (or dispose as the case may be) a large number of matters regarding which there is still much to be learned. As secretary of the organizing committee of the above association, Dr. Harrower will be pleased to hear from any of the readers of this journal who may be interested in learning more about the objects of this society and the advantages to them of membership in it.

While we think of it: How many of our readers would be interested in securing this series of articles when completed early next year in book form? Probably the cost would not be more than a dollar, and it has already been suggested to us that we publish them in more permanent form since the series of articles covers a very important phase of medicine on which there is very little literature convenient of access and to which frequent reference could be made with it. We should be glad to hear from those readers of the TEXAS MEDICAL JOURNAL who desire to have a book of this character, and if the demand is sufficient it certainly will be printed.

---

We take pleasure in introducing to our readers this month the following new firms:

A. A. Marks, New York.

The Pine Sanitarium, Chicago.

The Globe Metal Sign Works, Chicago.

The Phenadul Chemical Co., Boston, Mass.

Leo McDaniel, Cairo, Ill.

These firms have placed their advertisements in the TEXAS MEDICAL JOURNAL because they wanted to get in touch with the physicians of Texas and the great Southwest. Now, that they have been properly introduced, we hope that every reader of the JOURNAL will give them a good old Southern handshake, in the form of an order, and don't forget the new firms we introduced last month, and don't forget the dear old firms that have been with us, lo, these many years. They all help to make this JOURNAL a success; they carry their advertising here because they want your patronage. So don't forget them when you want anything in their line.

---

The careless sneezer is the great grip spreader.

---

The United States Public Health Service has trapped 615,744 rodents in New Orleans in the past eighteen months.

---

DO WE EAT TOO MUCH?—With much qualification, *men* incline to over-eating; *women,* large numbers, eat too little. I am in doubt as to how far this applies to women in every class of life. It certainly does to the middle and upper classes, and to married women in them with the care of households and the provision of food.—Sir James F. Goodhart, Bart., M. D., C. M., F. R. C. P. Lond., in *The Strand.*

